# Neurally dissociable cognitive components of reading deficits in subacute stroke

**DOI:** 10.3389/fnhum.2015.00298

**Published:** 2015-05-27

**Authors:** Olga Boukrina, A. M. Barrett, Edward J. Alexander, Bing Yao, William W. Graves

**Affiliations:** ^1^Language Behavior and Brain Imaging Lab, Department of Psychology, Rutgers, The State University of New JerseyNewark, NJ, USA; ^2^Stroke Rehabilitation Research, Kessler Foundation, West OrangeNJ, USA; ^3^Department of Physical Medicine and Rehabilitation, Rutgers-New Jersey Medical SchoolNewark, NJ, USA; ^4^Rocco Ortenzio Neuroimaging Center, Kessler Foundation, West OrangeNJ, USA

**Keywords:** MRI, lesion-deficit analysis, reading, semantics, phonology, orthography, stroke, aphasia

## Abstract

According to cognitive models of reading, words are processed by interacting orthographic (spelling), phonological (sound), and semantic (meaning) information. Despite extensive study of the neural basis of reading in healthy participants, little group data exist on patients with reading deficits from focal brain damage pointing to critical neural systems for reading. Here, we report on one such study. We have performed neuropsychological testing and magnetic resonance imaging on 11 patients with left-hemisphere stroke (<=5 weeks post-stroke). Patients completed tasks assessing cognitive components of reading such as semantics (matching picture or word choices to a target based on meaning), phonology (matching word choices to a target based on rhyming), and orthography (a two-alternative forced choice of the most plausible non-word). They also read aloud pseudowords and words with high or low levels of usage frequency, imageability, and spelling-sound consistency. As predicted by the cognitive model, when averaged across patients, the influence of semantics was most salient for low-frequency, low-consistency words, when phonological decoding is especially difficult. Qualitative subtraction analyses revealed lesion sites specific to phonological processing. These areas were consistent with those shown previously to activate for phonology in healthy participants, including supramarginal, posterior superior temporal, middle temporal, inferior frontal gyri, and underlying white matter. Notable divergence between this analysis and previous functional imaging is the association of lesions in the mid-fusiform gyrus and anterior temporal lobe with phonological reading deficits. This study represents progress toward identifying brain lesion-deficit relationships in the cognitive components of reading. Such correspondences are expected to help not only better understand the neural mechanisms of reading, but may also help tailor reading therapy to individual neurocognitive deficit profiles.

## Introduction

Reading is an important skill in contemporary society and its impairment as a result of brain damage represents a significant handicap. Yet, despite decades of research into the cognitive and neural mechanisms of reading, a basic question of what are the critical neural substrates supporting the cognitive components of reading remains unresolved. Our approach to this question is to use a theoretical framework from computational modeling which distinguishes three main components of reading: visual form (orthography), sound (phonology), and meaning (semantics). To link each reading component with its neural substrate, we tested reading in stroke patients and assessed their structural neuroimaging data with respect to lesion locations. The results presented here represent an important step toward filling a gap in current knowledge by qualitatively identifying potential critical neural substrates of the major cognitive components of reading.

Over the past several years, the application and relevance of computational models of reading (e.g., Seidenberg and McClelland, 1989; Plaut, 1996; Plaut et al., 1996) to neuropsychology has been gaining acceptance and validation. Consistent with the idea that reading relies on a multi-component functional neural network (Fiez and Petersen, 1998; Jobard et al., 2003; Indefrey and Levelt, 2004; Price, 2012), computational models posit that there are multiple cognitive mechanisms underlying lexical access. One of the computational models of reading which has received compelling support from computer simulations, behavioral, and neuropsychological data is the single-process triangle model (e.g., Seidenberg and McClelland, 1989; Plaut et al., 1996). It conceives of reading as an interactive activation of orthography, phonology, and semantics. This model has been useful in interpreting functional magnetic resonance imaging (fMRI) data (Fiez et al., 1999; Binder et al., 2005; Frost et al., 2005; Graves et al., 2010b), and has led to conceptual advances in understanding acquired reading disorders such as those arising from impaired semantic processing (Woollams et al., 2007).

According to the single-process model, the mapping between orthography (O) and phonology (P) develops as a function of frequency of exposure to different spelling-sound correspondence patterns in the course of learning to read. This process is mediated by semantics, such that the amount of semantic input depends on the nature of the word. Words that contain consistent spelling-sound correspondence patterns (e.g., -UST in DUST) can be pronounced without strong activation of semantic (S) content, whereas words that contain inconsistent spelling-sound correspondence patterns (e.g., -OST in HOST and COST) rely on access to meaning to a greater degree. According to the model, semantics is used to reduce interference between inconsistent O and P features of the input.

The computation of O, P, and S has been investigated behaviorally using lexical stimuli that vary in frequency, spelling-sound consistency, and imageability (e.g., Taraban and McClelland, 1987; Monsell, 1991; Strain et al., 1995; Jared, 2002). Frequency refers to how often a word occurs in print; consistency is measured as the difference between the number of “friends,” which share the rime pronunciation with the word, and the number of “enemies” with identically spelled but differently pronounced rimes (Seidenberg and McClelland, 1989); and imageability reflects the degree to which a word evokes an image. Imageability is often used as a measure of S processing because highly imageable words have richer, more easily computed S representations than less imageable words (Paivio, 1991; Plaut and Shallice, 1993). By applying the logic of the triangle model to lesion profiles of stroke patients and their behavioral performance on words that vary in frequency, consistency, and imageability, we can causally link O, P, and S processing with different parts of the neural network for reading. Additionally, we can begin to refine our knowledge of reading deficit profiles and syndromes in terms of impairments in one or more of these cognitive processes. For example, surface dyslexia is a syndrome in which patients are impaired in reading low-consistency words, but are unimpaired at reading high-consistency words, and non-words (pseudowords, pronounceable novel letter strings). This syndrome can be explained in terms of a S deficit (Woollams et al., 2007). A contrasting condition, acquired phonological dyslexia, involves impaired non-word reading (Coslett, 2000). The connectionist model offers a detailed account of this deficit centered on regions implicated in O–P mapping (Harm and Seidenberg, 1999). A recent review of data from 99 patients with stroke-induced aphasia and reading deficits highlighted the fact that the classical aphasia syndromes do not help to assess reading deficits as these can be co-morbid with all of the aphasia syndromes (Brookshire et al., 2014). By contrast, the approach used in the current study is to directly focus on critical neural systems underlying the cognitive components of reading.

To reveal the neural regions subserving the cognitive components of reading we conducted a lesion-deficit analysis. This approach establishes a relationship between damage to a circumscribed brain area and the resulting behavioral impairment (Damasio and Damasio, 2003). A lesion-deficit analysis by Hillis et al. (2001) is relevant in that they examined correlations between left-hemisphere areas of hypoperfusion during acute stroke and the presence of deficits affecting sub-components of reading aloud. Whether a particular component was impaired was determined using the pattern of patient performance on several lexical tasks. In the present study we focused on the reading components highlighted in the connectionist model, in contrast to the dual-route model applied by Hillis et al. (2001). We especially wanted to study the role of semantics in conversion of orthography to phonology, as the effect of semantics is often overlooked by the dual-route models.

In accordance with findings from functional neuroimaging of healthy participants, we expected that reading deficits would be associated with dysfunction of several left-hemisphere areas, including posterior middle temporal gyrus (pMTG) and posterior superior temporal gyrus (pSTG), angular gyrus (AG), supramarginal gyrus (SMG), mid-fusiform gyrus (FG), middle inferior temporal sulcus (ITS) and left opercular and triangular inferior frontal gyrus (IFG). The exact functions of some of these areas in language and reading are debated (e.g., Cattinelli et al., 2012; Danelli et al., 2013). For example, mid FG has been proposed to specifically process O word forms (Cohen et al., 2000, 2002; Vinckier et al., 2007; Glezer et al., 2009). Yet this area may also be sensitive to meaning (e.g., Devlin et al., 2006; Kherif et al., 2011), and there is evidence this area plays a role in the early mapping between O and P (e.g., Hillis et al., 2005; Graves et al., 2010b; Cattinelli et al., 2012; Mano et al., 2013). The AG has been implicated in processing semantics (e.g., Binder et al., 2009; Graves et al., 2010a; Price, 2012), although some have linked this area with reading ability through its putative role in supporting O–P conversion (e.g., Cao et al., 2008). Lesion-deficit findings should help clarify the extent to which these neuroimaging results relate to critical neural systems supporting the cognitive components of reading.

Following the logic of the triangle model, we tested for any processing components of reading that were impaired following stroke, using tasks that selectively relied on these components. The combination of O, P, and S scores from such tasks served as binary dependent variables identifying patients with and without the relevant deficits. In addition to using non-verbal (picture-based) tests, we measured how patients read words of varying frequency, imageability, and consistency as a way to obtain high-sensitivity criteria for degree of intact or impaired O, P, and S processing.

## Materials and Methods

### Participants

Data were collected from 11 patients (four males), mean age = 62.9 years (SD = 8.7), who were undergoing inpatient rehabilitation at the Kessler Institute for Rehabilitation. The inclusion criteria were: right-handedness prior to stroke, English as first language, left-hemisphere stroke within 5 weeks of study enrollment, and ability to carry out the experimental tasks. The exclusion criteria were: contraindication to magnetic resonance imaging (MRI; e.g., claustrophobia, pregnancy, extreme obesity, inability to lie flat, implanted ferromagnetic devices), uncorrectable hearing or vision difficulties, dementia, head trauma, tumor, multiple infarcts, severe psychiatric illness, and pre-stroke diagnosis of a reading or learning disability. Patients who indicated research interest upon admission were approached in the first week of being admitted to Kessler. Those who agreed and met the criteria were enrolled and typically tested in the second week of their stay. The patient’s demographic and neurological profiles are shown in **Table 1**.

**Table 1:** Psychometric and neurological data on the current patient sample.

Patient	Age	Gender	Years of education	GDS	BNT	HVLT immediate recall	COWA	Verbal fluency		Paraphasic errors


								Pace of Speech	Quantity of Words	
1	58	Female	16	18	15	22	8	1	1	Absent
2	68	Female	16	3	10	14	11	1	2	Semantic and phonemic
3	83	Male	12	1	14	16	42	1	2	Absent
4	61	Female	12	18	0	3	0	0	1	Phonemic
5	65	Male	12	15	3	5	0	1	1	Phonemic
6	56	Female	17	13	11	13	20	2	2	Absent
7	61	Male	18	2	9	13	13	1	1	Absent
8	69	Female	18	0	9	18	26	2	2	Absent
9	68	Female	12	2	10	14	8	1	2	Absent
10	59	Male	17	3	–	11	–	1	2	Absent
11	46	Male	16	11	11	13	9	1	1	Semantic and phonemic

*GDS, Geriatric Depression Scale (0–30; 0–9 normal; 10–19 mild to moderate depression; 20–30 severe depression); BNT, Boston Naming Test (short form 15 items); HVLT, Hopkins Verbal Learning Test (0–36); COWA, Controlled Oral Word Association (<15, non-fluent; >50, fluent); Pace of Speech (semi-quantitative scale of 0-2); Quantity of Words (semi-quantitative scale of 0–2)*.

### Tasks and Behavioral Data Acquisition

Patients underwent extensive psychometric testing, including the Florida Mental Status Exam (FMSE; Doty et al., 1990), Boston Naming Test (BNT; Kaplan et al., 1983), Geriatric Depression Scale (GDS; Yesavage et al., 1983), and a neurological exam administered by a licensed neurologist. Patient medical charts and all prior scans were also part of the dataset. The tasks designed to measure each of the cognitive components of reading included tests for phonological and semantic impairments using a touch screen version of the Pyramids and Palm Trees test (PPT; Howard and Patterson, 1992) adapted from Pillay et al. (2014) and a test for orthographic impairments using a two-alternative forced choice (task and stimuli adopted from Cassar and Treiman, 1997). All touchscreen tasks were preceded by practice sessions to ensure compliance with task instructions. The side of the target presentation was counterbalanced, such that the target appeared equally often on the left and right.

#### Semantic Tasks

**FIGURE 1:**
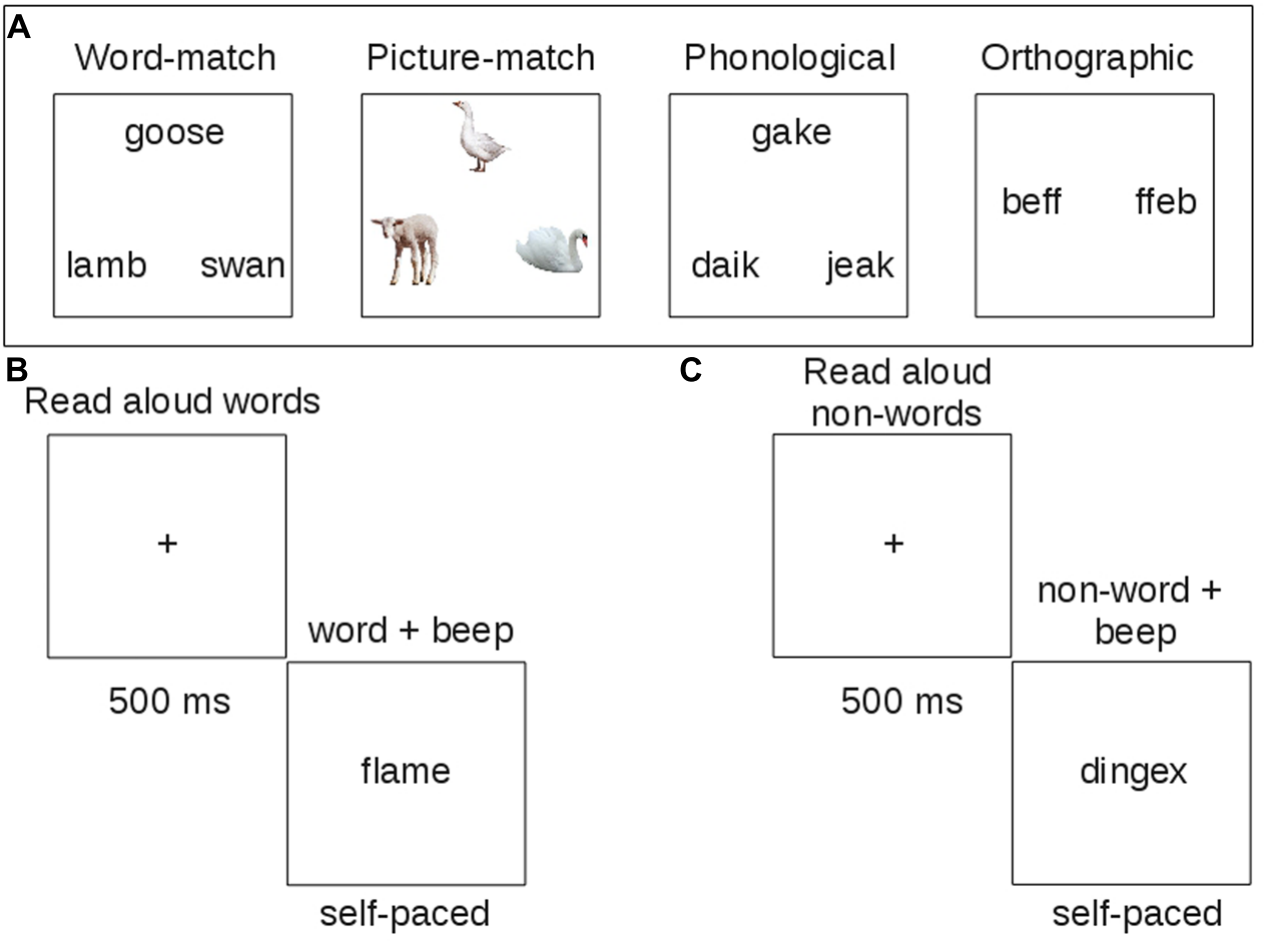
**Example trials of behavioral tasks administered to patients**. Each task was designed to measure one component of reading. **(A)** Touch-screen tasks; **(B)** reading aloud task with words; **(C)** reading aloud task with non-words.

On semantic trials, the target was a concrete noun presented at the top of the screen (see **Figure 1A**). The two choices at the bottom included a close semantic neighbor and a more distant semantic neighbor. Patients were instructed to select the word that was most similar in meaning to the target. The semantic task is administered once with words (word-matching) and once with pictures (picture matching), with the order of administration randomized. Word-matching items correspond to either abstract (20 trials) or concrete (40 trials) concepts. The concrete concepts were either living things (20 trials) or artifacts (20 trials). Trials also varied in difficulty, with higher feature similarity among all items on hard trials (30 trials). Picture matching items were color photographs representing concrete concepts from the living things category (34 trials) or artifacts (26 trials). Picture matching trials also varied in difficulty.

#### Phonological Task

On phonological tasks, all letter strings were pseudowords and the task was to select the string that rhymed with the target at the top. The choices included a close rhyme of the target with non-matching orthography and a close, but implausible rhyme.

#### Orthographic Task

In the orthographic task, all letter strings contained consonant doublets that are part of a trigram with high or low (position-constrained) frequency. Participants were asked to identify which string looked more like it could be a word.

#### Reading Aloud Tasks

Patients also completed a reading aloud task, where they read 120 words of high or low consistency, frequency, and imageability, and 60 non-words (i.e., pseudowords) matched to the words in length, and position-constrained bigram and biphone frequency (**Figures 1B,C**). Letter strings were presented simultaneously with a beep to orient patients’ attention to the string for reading aloud. The word stimuli were carefully matched across list conditions on length, orthographic and phonological neighborhood size (Orth N, Phon N), log bigram and biphone frequencies (Log BG, Log BP; see **Table 2**).

**Table 2:** Linguistic measures of word stimuli in the reading aloud task.

Word list	*N*	Length	Orth N	Phon N	Log BG	Log BP	Imag	Log F	Con
Low Imag, Low F, Low Con	15	4.33	7.00	15.80	2.97	2.68	3.76	0.37	0.33
Low Imag, Low F, High Con	15	4.53	7.07	15.20	2.99	2.63	3.75	0.34	17.00
Low Imag, High F, Low Con	15	4.33	7.00	13.87	3.10	2.69	3.80	1.87	0.47
Low Imag High F, High Con	15	4.60	7.33	13.40	2.98	2.68	3.80	1.82	17.53
High Imag, Low F, Low Con	15	4.33	7.53	14.27	3.05	2.70	5.94	0.32	0.40
High Imag, Low F, High Con	15	4.27	7.60	14.27	2.98	2.64	5.95	0.40	17.60
High Imag, High F, Low Con	15	4.47	7.33	16.47	3.08	2.71	5.96	1.83	0.53
High Imag, High F, High Con	15	4.47	7.33	14.67	2.99	2.64	5.93	1.84	17.33

*N, number of items; Orth N, Orthographic Neighborhood Density; Phon N, Phonological Neighborhood Density; Log BG, Log of Bigram Frequency; Log BP, Log of Biphone Frequency; Imag, Imageability Rating; Log F, Log of Word Frequency; Con, Consistency*.

### MRI Data Acquisition

The MRI data were collected at the Rocco Ortenzio Neuroimaging Center at the Kessler Foundation on a 3.0 Tesla Magnetom Skyra Scanner. High-resolution T1-weighted structural MRI scans (MPRAGE – Magnetization-Prepared Rapid Gradient-Echo) and T2 FLAIR (Fluid Attenuated Inversion Recovery) were acquired using a 20-channel Siemens head/neck coil. The MPRAGE was acquired with 1 mm^3^ isotropic voxels, TR = 2100 ms, TE = 3.43 ms, FoV = 256, flip angle = 9°, number of slices = 176. Axial T2 FLAIR images (1 mm × 1 mm in-plane resolution, 3 mm slice thickness) were acquired with TR = 9000 ms, TE = 91 ms, TI = 2500 ms, FoV = 256, flip angle = 150°, number of slices = 44.

### MRI Data Analysis

Magnetic resonance imaging data were preprocessed using FSL 5.0.6 software (FMIRB’s Software Library, http://fsl.fmrib.ox.ac.uk/fsl/fslwiki/). Images were skull stripped using BET (Smith, 2002), co-registered and normalized to the high-resolution MNI152 1 mm^3^ standard space image using FSL FLIRT software (Jenkinson and Smith, 2001; Jenkinson et al., 2002) and the lesion-filling utility (Battaglini et al., 2012), where all lesioned voxels were filled in with gray values of similar intensity to the surrounding tissue to improve registration. Lesion tracing was done using Fslview software in the original MPRAGE space by comparing overlays of the MPRAGE and FLAIR images. Lesion masks were generated by filling in the tracings and were then transformed into standard space by applying the same transformation matrix as was used to normalize the MRAGE images. Anatomical labels were derived from the Harvard-Oxford cortical and subcortical structural atlases, JHU ICBM-DTI-81 white-matter labels, and JHU white-matter tractography atlas provided as part of the FSL 5.0.6 software.

## Results

### Behavioral Results

With the exception of one participant with severe alexia, all participants completed the semantic word-matching task. Due to a software error only responses on half of the S word-matching and picture-matching trials were recorded. The average number of trials correct on the word-matching task was 23.40 (SD = 6.08) out of the 30 recorded. Three patients produced scores that were at chance for choosing either the correct target or the distractor. This was decided according to the binomial probability test, where chance probability was equal to 0.5 (probability of choosing one option in a 2-alternative forced-choice paradigm). Overall, patients completed the S picture-matching task with an average accuracy of 25.23 (SD = 3.90) trials correct out of 30. One patient, who performed poorly in the S word-matching task, also performed at chance on the S picture-matching task.

In the O task, overall patient accuracy was 48.6 items out of 60 (SD = 10.4) and only one patient failed to perform significantly better than chance. In the P task, patients correctly answered 41.5 trials out of 60 (SD = 10.0). Five patients failed to perform above chance. To ensure unbiased sampling, we subsampled P and O trials to match the number of S trials. This resulted in the same patients being identified as performing below chance as in the full set of trials. Overall reading impairment profiles of our patient sample are shown in **Table 3**. Spatial distribution of responses on the left and the right side of the screen was balanced for 9 out of 11 participants. One patient showed a slight rightward bias (109 out of 180 responses on the right) and another patient showed a slight leftward bias (60 out of 180 responses on the right). This bias was due to the patients’ performance in the S tasks.

**Table 3:** Summary of reading impairments in the current patient sample.

Patient	S impairment	O impairment	P impairment
1			
2	+		+
3			
4	+	+	+
5	+		+
6			
7			
8			
9			
10			+
11			+

A cross indicates near chance performance and therefore presence of an impairment in a given cognitive component.

**FIGURE 2:**
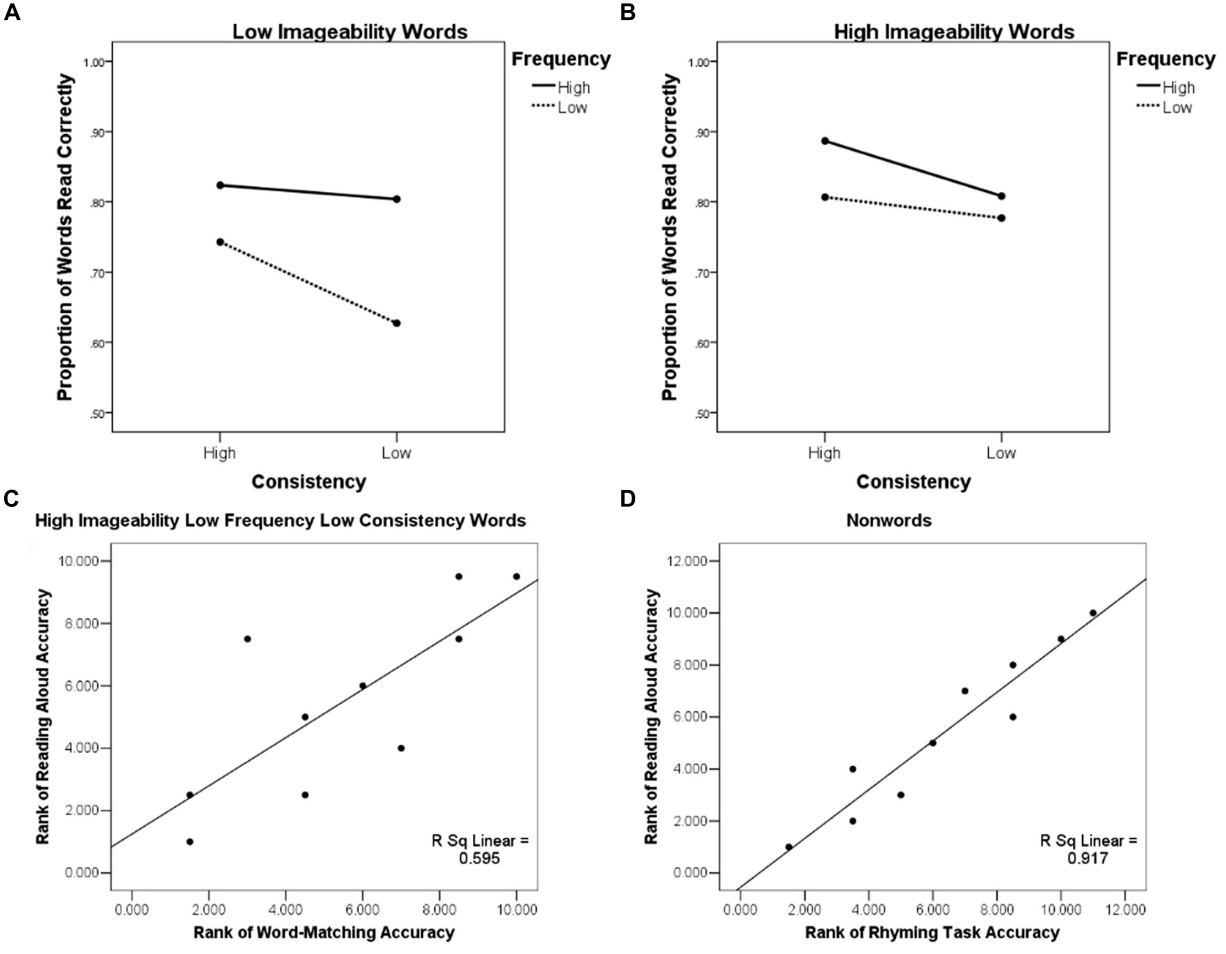
**Behavioral effects illustrating the role of semantics in reading aloud: **(A)** Patient accuracy in the reading aloud task as a function of word imageability, frequency, and consistency showing a three-way interaction of these variables, low imageability words; **(B)** Patient accuracy in the reading aloud task as a function of word imageability, frequency, and consistency showing a three-way interaction of these variables, high imageability words; **(C)** Rank-rank association scatter plot of patients’ accuracy in reading aloud words of high imageability low frequency and low consistency and their performance on the word-matching (S) task; **(D)** Rank-rank association scatter plot of patients’ accuracy in reading aloud non-words and their performance on the rhyming (P) task**.

We also examined patient reading aloud performance. One patient’s reading performance could not be measured because this patient dropped out of the study. In the other 10 patients, a three-way ANOVA with high and low levels of imageability, frequency, and consistency revealed an effect of imageability [*F*(1,9) = 8.87, *p* < 0.05], frequency [*F*(1,9) = 16.11, *p* < 0.005], and consistency [*F*(1,9) = 8.33, *p* < 0.05], with better reading for high (*M_correct_* = 0.82, SD = 0.24) than low-imageability (*M* = 0.75, SD = 0.26); high- (*M* = 0.83, SD = 0.25) than low-frequency (*M* = 0.74, SD = 0.25); and high-(*M* = 0.82, SD = 0.26) than low-consistency (*M* = 0.76, SD = 0.25) words. Additionally, a significant two-way interaction of frequency and imageability [*F*(1,9) = 5.60, *p* < 0.05], and a three-way interaction of frequency, imageability, and consistency [*F*(1,8) = 7.00, *p* < 0.05] emerged. The three-way interaction reflected the fact that patients were less accurate at reading low frequency, low consistency words when they were of low imageability (**Figure 2A**). When imageability was high, the low-consistency, low-frequency words were read with accuracy similar to other words (**Figure 2B**).

Individual performance profiles showed that the same patients who were impaired in the picture and word-matching tasks (2, 4, 5) also had the lowest accuracy (0.57; 0.17; and 0.57, respectively) when reading aloud words of low frequency and low consistency in our sample of patients (see **Figure 2C**). The patients who were impaired in the rhyming task (2, 4, 5, 10, 11) were also the same patients that had the lowest performance accuracy in non-word reading (0.33, 0.23, 0.42, 0.52, 0.20, respectively; see **Figure 2D**). These findings were investigated with a series of regression analyses.

Accuracy on word-matching and picture-matching S tasks was positively associated with the accuracy of reading aloud for words [*F*(2,7) = 6.35, *p* < 0.05] and non-words [*F*(2,7) = 13.99, *p* < 0.005], as indicated by a regression analysis with word-matching and picture-matching accuracies as predictors and reading aloud accuracy for words or non-words as an outcome measure. However, when age, years of education, and the GDS score (see **Table 1**) were entered as covariates, word-matching and picture-matching performance no longer predicted word or non-word reading accuracy. Accuracy in the P task predicted non-word reading accuracy [*F*(4,5) = 15.43, *p* < 0.01], but not word reading accuracy (*p*> 0.05).

Accuracy of O task performance predicted word reading accuracy [*F*(4,5) = 8.17, *p* < 0.05] and only marginally predicted non-word reading accuracy [*F*(4,5) = 4.69, *p* = 0.06]. The association between S task performance and reading aloud accuracy for words of low frequency and consistency is predicted by the connectionist reading model. Although our findings are consistent with this prediction, in lieu of the regression results, they are considered preliminary and await replication in a larger sample of patients.

In our study the responses were self-paced and therefore a speed/accuracy trade-off (i.e., increased accuracy with longer responses) was a potential issue that needed to be addressed. Importantly, the accuracy data were sensitive to the deficit profiles or our participants and did not show either a floor or a ceiling effect. To test if our accuracy and reaction time (RT) data showed a tradeoff, we looked at the correlation between these two measures across patients. If the patients who were impaired in accuracy on a given task were also slower to perform the task, we would expect a negative correlation between RT and accuracy. In contrast, if patients were boosting their accuracy by taking longer to complete the task, we would see a positive correlation between these two measures. To prepare the RT data for testing between these possibilities, responses more than 3 SD from the mean were removed. This resulted in the removal of 1.02% of the data from the picture matching task, 4.15% of the data from the word matching task, and 3.23 and 4.2% of the data from the O and P tasks, respectively. After the data were trimmed we ran 4 Pearson correlations between RT and accuracy (one for each task). 3 of the correlations were significant in the predicted direction. Specifically, for the word matching task, *r* = -0.76, *t*(9) = -3.46, *p* < 0.01; for the picture matching task, *r* = -0.75, *t*(10) = -3.57, *p* < 0.01; and for the O task, *r* = -0.77, *t*(10) = -3.82, *p* < 0.005. For the P rhyming task, the correlation was also in the negative direction, but did not reach significance, *r* = -0.34, *p* = n.s. Thus, we show that across all tasks there is either strong evidence against speed-accuracy tradeoffs, or no evidence for them.

### MRI Results

**FIGURE 3:**
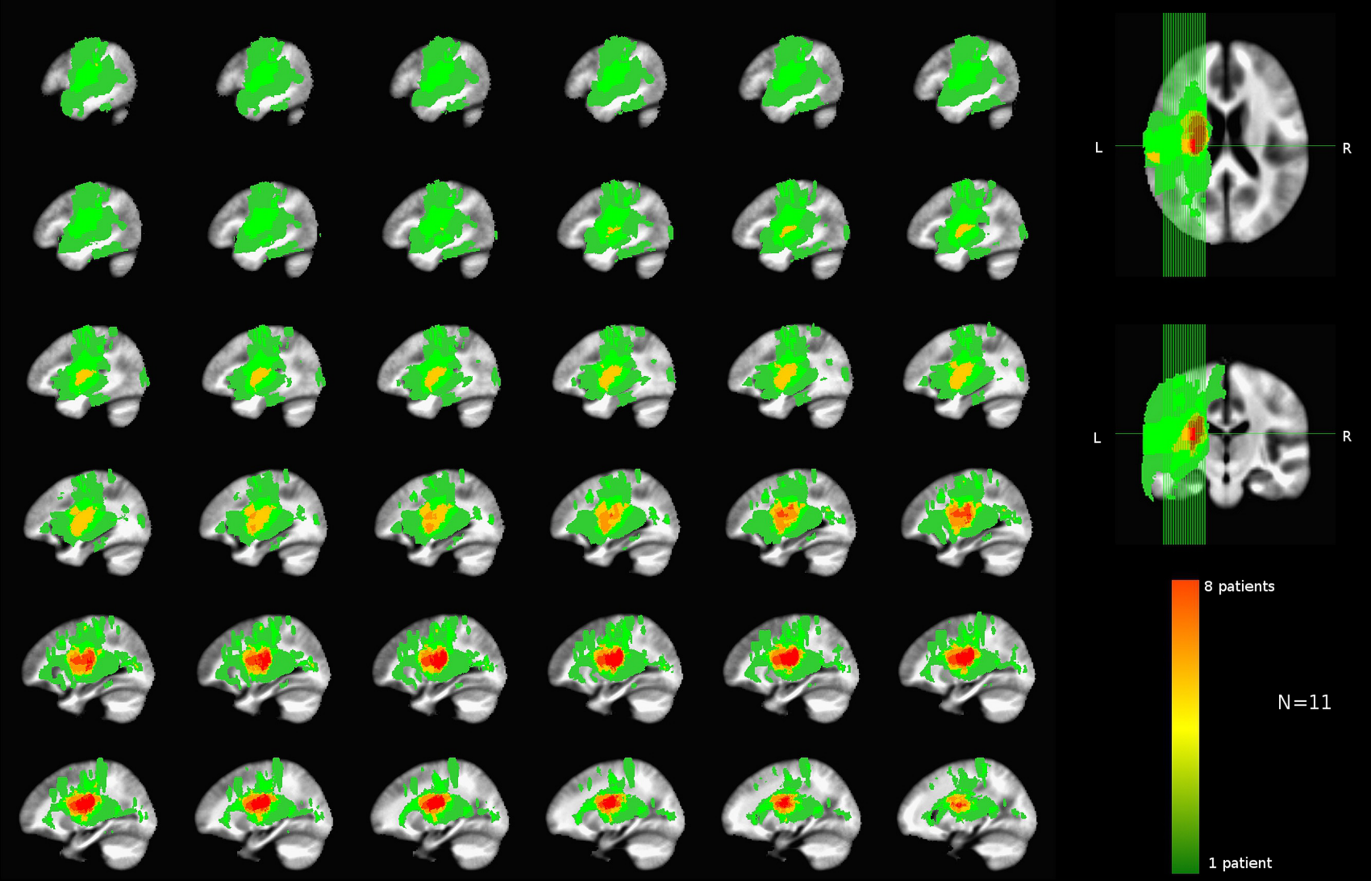
**Group lesion coverage map of the left hemisphere. All patients were included in the map, however, the greatest number of patients with overlapping lesions was eight as indicated on the color bar**.

Of the 11 total patients, 10 have undergone the MRI scans described above. For one patient a comparable set of clinical MRI scans was used that was acquired prior to a heart monitor implant, which had not been tested for safety at 3T. Hence the data were aggregated from clinical and research scans. The list of left-sided lesion locations identified for each patient is shown in **Table 4**. A group lesion coverage map is shown in **Figure 3**. Overall, lesions fell primarily in the middle cerebral artery territory and covered medial temporal, frontal, parietal and occipital cortices, middle temporal cortex, basal ganglia, inferior frontal and superior parietal cortices, as well as the underlying white matter.

**Table 4:** The left-sided lesion locations identified for each patient.

Patient	Lesion sites
1	Superior frontal gyrus (SFG) extending to postcentral gyrus (PCG) and anterior cingulate cortex (ACC)
2	Corona radiata (CR)
3	Dime-size lesions in frontal, parietal, and occipital lobes
4	Parietal lobe lesion extending to posterior cingulate cortex (PCC), parietal operculum (PO), and the insula
5	Basal ganglia (putamen and globus pallidus), thalamus, and internal capsule (IC)
6	Basal ganglia (caudate nucleus), CR, superior fronto-occipital fasciculus (SFOF), and anterior IC
7	Basal ganglia (caudate nucleus) and surrounding white matter including CR, SFOF, and anterior IC
8	Basal ganglia (putamen), superior CR, SFOF, and anterior IC
9	Thalamus and basal ganglia (caudate nucleus)
10	Brain stem, posterior FG, and superior parietal lobule
11	Inferior frontal gyrus (IFG) and inferior fronto-occipital fasciculus (IFOF), IFG pars opercularis, superior longitudinal fasciculus (SLF), parietal operculum, planum temporale, AG, SMG, lateral occipital cortex, and anterior temporal cortex.

#### Lesion-Deficit Associations

Qualitative maps of lesion-deficit associations were created by subtraction of lesion maps for patients who were at chance on the touch-screen task for a given reading component (S, O, and P), minus patients with other component impairments and patients without the relevant deficits. For example, for lesion-deficit associations in phonological processing, all lesioned voxels (3D brain pixels) for patients with chance performance on the rhyming task were combined into a binary map. A similar binary map was also created for patients who were at chance on the S tasks. As only a single patient was found to have an O deficit, the lesion mask of that patient served as the binary map for orthographic processing. A subtraction was then carried out between the P and the S maps and between the P and O maps. Next, voxels remaining in both subtractions (a logical AND) were saved to an intermediate P map. As a final step, we subtracted from the intermediate P map all of the lesioned voxels common to the unimpaired participants. This multi-step estimation of lesioned P voxels was performed to identify areas specific to P processing deficits in that they were neither associated with any other measured deficit, nor were they found in unimpaired patients. The analogous procedure was followed for estimating lesion-deficit associations for S and O processing.

**FIGURE 4:**
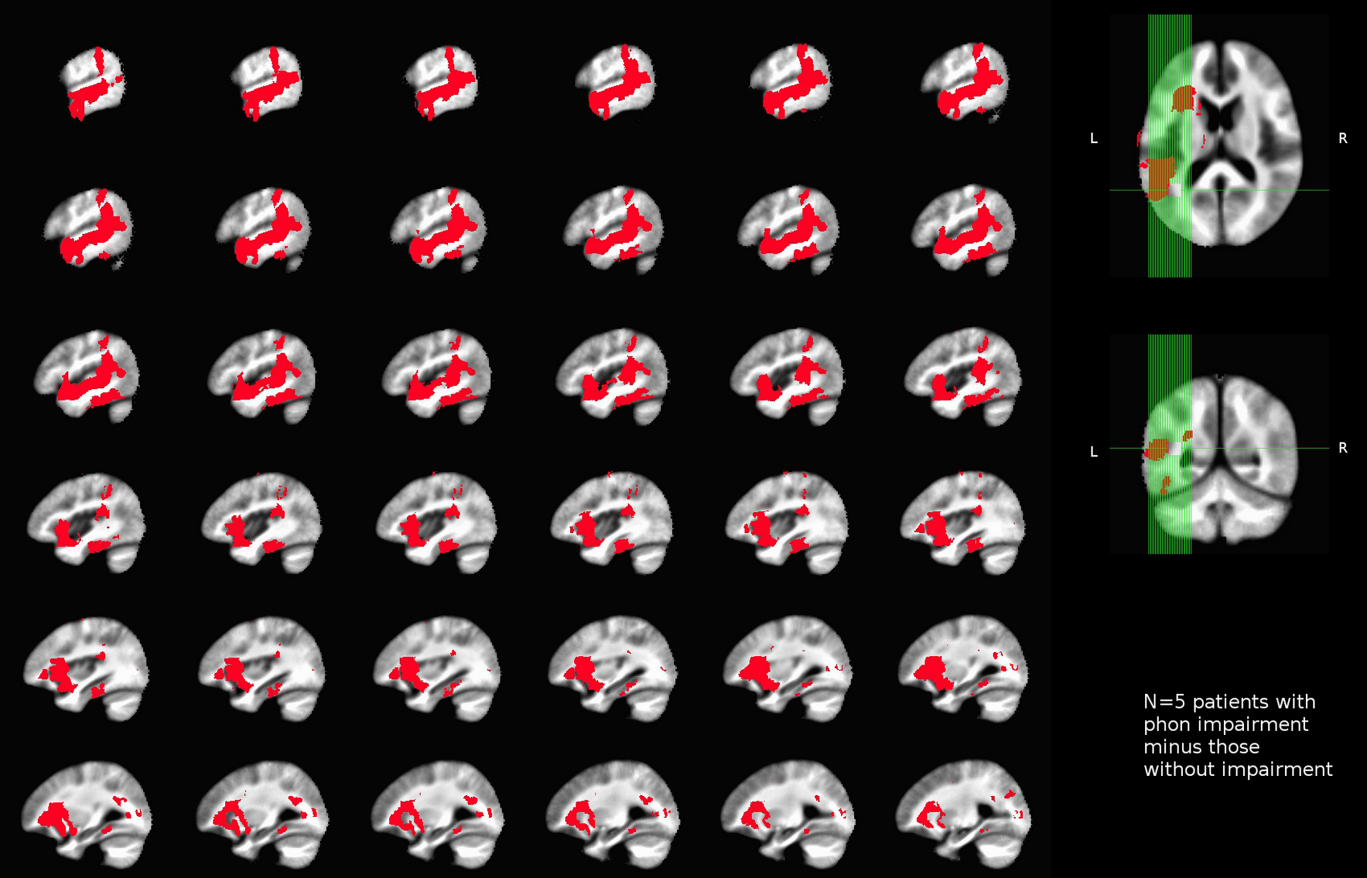
**Unthresholded map of areas uniquely associated with impaired performance on the phonological task (rhyming) in our sample of left-hemisphere stroke patients**.

No areas were uniquely associated with O deficits, as we only had one patient showing chance performance in our O touch-screen task, and this patient also had impairments in S and P components. Similarly, semantic deficits were identified in three patients (2, 4, and 5), with two (2, 5) patients presenting with a concurrent P impairment, and one patient (4) with concurrent O and P impairments. After a subtraction procedure no areas were uniquely associated with semantic processing. Our main finding was that we were able to identify areas uniquely associated with phonological deficits. Poor performance on the phonological task was associated with damage to a number of areas, including superior temporal gyrus, parietal operculum (PO) and insula, planum polare, and planum temporale (PT; i.e., primary and association auditory cortices), temporal and temporo-occipital fusiform cortex and ITS, SMG, pMTG, temporal-polar regions, and the pars opercularis of the IFG. Among white matter tracts associated with P deficits were IFOF, forceps major, anterior thalamic radiation, anterior corona radiata, uncinate fasciculus, and SLF (**Figure 4**).

## Discussion

This lesion-deficit study of patients with sub-acute left-hemisphere stroke revealed several findings. One general pattern was that left-sided supra-tentorial cortical and subcortical lesions were often associated with reading impairments. These reading deficits occur along orthogonal cognitive measures, including those that rely on semantic, orthographic, and phonological processing. An important finding from this work was that tests aimed at cognitive sub-components of reading can lead to identification of dissociable reading deficit profiles, even in a relatively small sample of stroke patients. Moreover, deficits identified using these sub-components do not directly correspond to known aphasia sub-types, as some of our patients diagnosed with non-fluent aphasia performed well across all reading measures (e.g., patient 1; suggesting at least some degree of intact phonological processing) while others without a diagnosis of aphasia nonetheless showed impaired reading (e.g., patient 10, who had a phonological deficit). This observation is consistent with earlier work in aphasia. As early as the 1920s, Henry Head noted that (1) clinical symptoms in aphasia could be grouped under a general term of “symbolic thinking and expression”; (2) that each clinical case of aphasia is the result of loss of one or more of such groups of functions, and (3) that careful analysis was necessary to identify the basic set of functions that were impaired or spared in an aphasic patient (Head, 1921). The work of Schwartz (1984) highlighted the notion that functional dissociations can cut across aphasia categories, while Marshall and Newcombe (1973) noted that presence of the three major types of acquired dyslexic errors, visual, semantic, and grapho-phonological, was often dissociated from aphasia symptoms. Most recently, Brookshire et al. (2014) presented a literature review showing that reading deficits are often found in all aphasia sub-types and that there is not a one-to-one association between aphasia and reading deficits.

An approach adapted in this work was to utilize one of the dominant computational models of reading, the triangle model (Seidenberg and McClelland, 1989; Plaut, 1996), for identification of impairments along processing components that are important for reading. Our behavioral data revealed that reading aloud performance changes as a function of the major cognitive subcomponents of reading. The division of labor among pathways connecting orthographic, phonological, and semantic representations during reading has been shown to be modulated by lexical factors such as word frequency, spelling-sound consistency, and imageability (Plaut et al., 1996; Harm and Seidenberg, 2004). Specifically, a three-way interaction among these variables is predicted by the dynamics of the triangle model and suggests that relying on S processing can improve reading performance on words with less straightforward O–P mappings. We found that patients showed poor accuracy when reading aloud low-frequency and low-consistency words, but performance on these kinds of words was rescued when they were of high imageability. Moreover, the patients most impaired in S processing also presented with the worst reading accuracy for low-frequency and low-consistency words. This pattern is consistent with prior behavioral findings in healthy readers showing that high word imageability facilitates reading aloud (e.g., Strain et al., 1995; Lichacz et al., 1999), and with neuroimaging literature showing that areas associated with semantic processing increase their influence on areas associated with phonological processing during reading of low-frequency, high-imageability words (Boukrina and Graves, 2013). In addition, this finding is in line with patient literature showing that semantic dementia, a relatively selective impairment of semantic information due to neurodegeneration (Hodges and Patterson, 1997), is associated with impaired reading of low-frequency and low-consistency words (Woollams et al., 2007). In our study we recorded RTs on each trial. As RT is a continuous measure, there is no straightforward way of assessing whether a patient was impaired in speed of processing, short of comparing their RTs to a matched comparison group with a different type of brain injury. We have specific plans to do this in the future, but currently such data are not available. Absent this, the possibility remains that deficits may be present in terms of reduced speed of response (or conversely, increased time to respond) on a given task, and such deficits would not be detected by relying on response accuracy alone. Importantly, however, our RT data co-patterned with accuracy showing that poor accuracy was typically accompanied by slower response time. Moreover, we did not observe ceiling or floor effects in accuracy. These findings suggest that both accuracy and RT measures were sensitive to impairments and that they were mutually consistent.

For our neural findings, using a strict subtraction analysis we were able to identify areas that were uniquely associated with deficits in phonological processing as measured by a rhyming task. For semantic and orthographic deficits, no unique associations between lesion locations and deficits could be made. We expected that semantic processing would be linked with areas, such as the precuneus, AG, and the underlying white matter previously implicated in semantic processing by healthy readers using functional neuroimaging (Binder et al., 2009; Price, 2012). Other potential brain areas often associated with semantic processing using functional neuroimaging are, for example, IFG *pars triangularis* (Poldrack et al., 1999; Bookheimer, 2002; Jobard et al., 2003; Binder et al., 2009) and inferior temporal sulcus (Binder et al., 2009; Graves et al., 2010b, 2014). In our analysis these areas were not found to be associated with semantic deficits. One important caveat of our findings is that the discovery of lesion-deficit associations was limited by the relatively small number of patients enrolled, which led to limitations on the distribution of deficits (e.g., we have been unable to identify sub-groups of patients with unique semantic and orthographic deficits), and limits on the area of lesion coverage. For example, parts of the prefrontal and anterior temporal cortices were not found in our lesion set. We therefore cannot speak to the possible association of these areas with impaired cognitive components of reading. Some parts of the anterior temporal lobe, however, were included in the current lesion coverage, but were associated with phonological processing, as discussed next.

Among the areas found in this study to support phonological processing were areas of the anterior temporal lobe (ATL; including the planum polare and anterior superior temporal gyrus), the PT, pSTG, SMG, MTG, PO, insula, ITG and mid FG, and opercular IFG. Of these, the PT, pSTG, SMG, MTG, PO, and opercular IFG have all been consistently associated with aspects of phonological processing. For example, the PT and pSTG are associated with phonological processing in word (Indefrey and Levelt, 2004; Graves et al., 2007) and non-word (Graves et al., 2008) production. In addition, the posterior MTG and SMG have been associated with phonological processing in reading with fMRI in typical readers (e.g., Jobard et al., 2003; Vigneau et al., 2006; Graves et al., 2010b; Cattinelli et al., 2012) and with underactivation in readers with dyslexia compared to typical readers (Maisog et al., 2008; Richlan et al., 2009). Disrupted phonological processing is also a key deficit in Wernicke’s aphasia, which is associated with damage to the PT, pSTG, and SMG (Damasio, 1998; Hickok and Poeppel, 2007; Buchsbaum et al., 2011). This is relevant to reading not only because of the involvement of phonology, but also because an asymmetry in the size of the left compared to right PT has been linked with reading deficits in developmental dyslexia (e.g., Morgan and Hynd, 1998). The IFG, including its pars opercularis, has also been consistently associated with phonological processing, both in previous studies of healthy participants (Poldrack et al., 1999; Bookheimer, 2002; Vigneau et al., 2006) and in patients with acquired reading deficits (Fiez et al., 2006; Rapcsak et al., 2009).

In addition to the relatively straightforward findings just discussed, a somewhat surprising finding was the association of temporo-occipital fusiform cortex, including the mid-FG location of the putative visual word form area (VWFA), with phonological and not orthographic deficits. In our P task, patients were required to convert letters into their phonemes in order to make a rhyming judgment, and lesions in mid-FG and surrounding cortices were associated with impairments on this task, but not on a task requiring knowledge of simple bigram distribution statistics of English (our O task). Previous functional neuroimaging and patient studies have suggested that the putative VWFA supports recognition of orthographic word forms (e.g., Cohen et al., 2000, 2002; Gaillard et al., 2006; Vinckier et al., 2007; Glezer et al., 2009). However, other recent evidence from reading aloud in healthy participants suggests a more general role of the mid-FG in reading (Graves et al., 2010b). Activity in this area has been implicated in phonological decoding (Schurz et al., 2010), acquisition of orthography-phonology mappings (Brem et al., 2010; Striem-Amit et al., 2012), and co-activation of orthography and phonology (Yoncheva et al., 2010; Mano et al., 2013). Its dysfunction has further been linked with developmental dyslexia (Richlan et al., 2010). Therefore, our finding of phonological processing in the VWFA is consistent with previous evidence, and it speaks to the debate regarding the specific nature of neuro-cognitive processing in this area, contrasting with accounts of orthography-specific processing in the VWFA that do not include phonology (e.g., Glezer et al., 2009, 2010). The current state of controversy over the function of this area is nicely illustrated by Sebastian et al. (2014) where the authors argue, based on evidence from lesion-deficit analysis in 234 patients with left-hemisphere stroke, that the role of the VWFA is the “computation of word-centered grapheme description, independent of size, font, location, or orientation, critical for reading and spelling” (p. 513). Our results support the interpretation of the function of the mid-FG as also critical for phonological processing, and highlight the need to assess the functional sub-components of reading in order to determine structure-function associations.

Lesions to the ATL were also found here to be related to phonological rather than semantic deficits. That is, at least one patient with damage to the ATL from stroke showed intact semantic and orthographic but impaired phonological processing. While this result awaits replication in a larger group study, it suggests that, at least in principle, damage to the ATL from stroke need not significantly impair semantic processing, but may instead degrade phonological processing. Although this finding runs counter to the pattern seen in patients with semantic dementia (Patterson et al., 2007), it is consistent with other large-scale lesion-deficit studies that used a combination of picture naming and identification to distinguish semantic from phonological processing. Specifically, patients with focal lesions to the left ATL were able to identify pictures of concrete entities, but could not name them, suggesting a phonological rather than semantic deficit (Damasio et al., 1996, 2004). However, the comparison is not entirely straightforward, as their ATL deficit findings were relatively selective for retrieving famous proper names.

When considering both the performance and brain lesion data, we note that phonological impairments were the ones most commonly seen in our patients, with nearly all patients having difficulty reading aloud pseudowords. This may be due at least in part to pseudoword reading being a relatively difficult task, making performance on this task vulnerable to several possible sources. It may be for this reason that pseudoword reading was correlated with performance on the semantic tasks, despite pseudowords lacking meaning and therefore presumably not relying on semantic processing. That is, a phonological deficit would certainly impair pseudoword reading aloud, but this could also occur with a semantic processing deficit if that deficit also impinged on a third, perhaps domain-general function like verbal working memory that had widespread implications for reading. Such an account would be consistent with the apparently domain-general reading deficits reported in the study of acquired phonological dyslexia and dysgraphia by Rapcsak et al. (2009).

Lastly, we found that white matter lesions co-occurred with phonological processing deficits. Although we did not conduct a formal analysis of white matter integrity, such as one that relies on diffusion tensor imaging (DTI), such analysis represents a future direction in our study. Preliminary evidence points to several white matter tracts that may support the phonological processing component of reading tested here. For example, we identified the uncinate fasciculus, ILF, IFOF, forceps major, anterior thalamic radiation, anterior CR, and SLF as potentially important for phonological processing. Recent reviews of DTI and reading studies link some of these areas with reading ability. For example, a review by Ben-Shachar et al. (2007) pointed to the association of processing along CR, callosal fibers, and SLF with reading problems in dyslexia. However, it did not link these fiber tracts with deficits in specific sub-components of reading, as was the goal in the current study. A meta-analysis by Vandermosten et al. (2012) synthesized a large body of evidence from healthy and dyslexic readers and proposed two major information transfer routes for reading: the ventral orthographic stream and the dorsal phonological stream. The orthographic stream consists of the ILF and the IFOF, with a role in both orthographic and semantic processing. The phonological stream consists of the arcuate fasciculus (AF) and the SLF. Thus, some of our findings such as the association of CR and SLF with reading and phonological processing are consistent with the DTI literature on reading.

Several of the white matter tracts we identified as crucial for phonology (e.g., uncinated fasciculus, IFOF) have been implicated in semantic processing. The uncinate fasciculus was thought to be a part of a pathway (connecting temporal lobe and inferior frontal regions) which subserves semantic processing (Vigneau et al., 2006). Similarly, left IFOF was previously found (using structural and functional connectivity measures) to link areas such as left posterior MTG and the orbital part of IFG (Turken and Dronkers, 2011), both of which were identified as areas supporting comprehension (Dronkers et al., 2004). Dronkers et al. (2004) conducted a lesion-deficit mapping study with 64 chronic left-hemisphere stroke patients and found that both left posterior MTG and left orbital IFG were associated with deficits in selecting a picture that accurately represented the meaning of a spoken sentence. Posterior MTG was associated with impairments in understanding a number of different sentences, and the authors suggested that it subserves comprehension on a single-word level. However, auditory comprehension tasks, such as the one used by Dronkers et al. (2004), require both P and S processing and the pattern of deficits observed in their study may have stemmed from the impairments along either component, or even the P component alone. The approach demonstrated here has so far not been widely used to investigate the neural basis of acquired reading deficits. This is surprising considering its potential usefulness as a way of defining language and reading impairments in terms of specific deficits. This in turn may facilitate the development of targeted, effective treatments.

The results of our lesion-deficit analysis are broadly consistent with prior neuroimaging literature showing associations of superior and middle temporal cortex, superior parietal and inferior frontal cortex with P processing. Our findings also help shed light on neural processing in areas that are thought to be functionally specialized for particular components of reading. For example, a large part of cortex in mid-FG, including the putative VWFA, in our study was associated with P processing. Together with recent neuroimaging findings, our lesion-deficit results point to a role for mid-FG in integrating orthography and phonology. Similarly, some findings in white matter tracts previously thought to underlie auditory comprehension (uncinate fasciculus and ILF) were more precisely linked with P processing using our information processing approach. While the present study includes a relatively small number of patients, we believe that our results offer a valuable contribution to the literature, for a number of reasons. First, the number of participants is within the range of recently published lesion-deficit studies of reading; second, our pattern of findings speaks to currently debated issues in the field (such as the role of the anterior temporal lobe, the putative VWFA, and of the specific white matter tracts in reading); and third, its relevance to patients and patient-centered research increases the practical significance and urgency of dissemination of this work. Our study also demonstrates the feasibility of applying a novel information processing framework to the assessment and remediation of acquired reading deficits following left-hemisphere stroke, which, ultimately, can add to our understanding of brain processes supporting skilled and impaired reading.

## Conclusion

This work demonstrates the feasibility of a group-level lesion-deficit study of the cognitive components of reading. Such studies are surprisingly rare, and stand to advance our understanding of the cognitive neuroscience of reading. In addition, such studies may provide an important link between the basic cognitive neuroscience of reading and the ability to tailor post-stroke therapies to particular reading deficits.

## Conflict of Interest Statement

The authors declare that the research was conducted in the absence of any commercial or financial relationships that could be construed as a potential conflict of interest.
